# Ethanol Electro-Oxidation on Catalysts with S-ZrO_2_-Decorated Graphene as Support in Fuel Cell Applications

**DOI:** 10.3390/nano12193327

**Published:** 2022-09-24

**Authors:** Maryam Yaldagard, Mehrdard Shahbaz, Hyoun Woo Kim, Sang Sub Kim

**Affiliations:** 1 Department of Chemical Engineering, Faculty of Engineering, Urmia University, Urmia 5766-151818, Iran; 2 Department of Materials Science and Engineering, Faculty of Engineering, Urmia University, Urmia 5766-151818, Iran; 3 Division of Materials Science and Engineering, Hanyang University, Seoul 04763, Korea; 4 Department of Materials Science and Engineering, Inha University, Incheon 22212, Korea

**Keywords:** ethanol electro-oxidation, electrocatalyst, ZrO_2_, graphene, fuel cell

## Abstract

Direct ethanol fuel cells (DEFCs) are considered the most suitable direct alcohol fuel cell (DAFC) in terms of safety and current density. The obstacle to DEFC commercialization is the low reaction kinetics of ethanol (C_2_H_5_OH) oxidation because of the poor performance of the electrocatalyst. In this study, for the first time, graphene nanoplates (GNPs) were coated with sulfated zirconium dioxide (ZrO_2_) as adequate support for platinum (Pt) catalysts in DEFCs. A Pt/S-ZrO_2_-GNP electrocatalyst was prepared by a new process, polyol synthesis, using microwave heating. Field emission scanning electron microscope (FESEM) imaging revealed well-dispersed platinum nanoparticles supported on the S-ZrO_2_-GNP powder. Analysis of the Fourier transform infrared (FTIR) spectrometry confirmed that sulfate modified the surfaces of the sample. In X-ray diffraction (XRD), no effect of S-ZrO_2_ on the crystallinity net in Pt was found. Pt/S-ZrO_2_-GNP electrode outperformed those with unsulfated counterparts, primarily for the higher access with electron and proton, confirming sulfonating as a practical approach for increasing the performance, electrocatalytic activity, and carbon monoxide (CO) tolerance in an electrocatalyst. A considerable decrease in the voltage of the CO electrooxidation peak from 0.93 V for Pt/C to 0.76 V for the Pt/S-ZrO_2_-GNP electrode demonstrates that the new material increases activity for CO electrooxidation. Moreover, the as-prepared Pt/S-ZrO_2_-GNPs electrocatalyst exhibits high catalytic activity for the EOR in terms of electrochemical surface area with respect to Pt/ZrO_2_-GNPs and Pt/C (199.1 vs. 95 and 67.2 cm^2^.mg^−1^ Pt), which may be attributed to structural changes caused by the high specific surface area of graphene nanoplates catalyst support and sulfonating effect as mentioned above. Moreover, EIS results showed that the Pt/S-ZrO_2_-GNPs electrocatalyst has a lower charge transfer resistance than Pt/ ZrO_2_-GNPs and Pt/C in the presence of ethanol demonstrating an increased ethanol oxidation activity and reaction kinetics by Pt/S-ZrO_2_-GNPs.

## 1. Introduction

Direct alcohol fuel cells (DAFCs) are energy conversion devices that directly change the chemical energy of alcohol into electricity via electrocatalytic reactions [1]. Recently, DAFCs have been considered an alternative to standard internal combustion engines for their prospective usage in compact electronic systems and transportation [2,3,4,5,6,7]. Furthermore, as liquid fuels, low-molecular-weight alcohols have superiority over pure hydrogen; with slight modifications, they can be reserved and moved easily via the existing gasoline infrastructure [8,9,10]. Without an external reformer, DAFCs use ethanol or methanol directly as a fuel, simplifying them and reducing their size.

Ethanol has a greater power density than methanol (8.01 vs. 6.09 kWh. kg^−1^) [11]. Furthermore, ethanol has a lower price, toxicity, and crossover effect with solid electrolytes like a Nafion membrane [12,13,14], so it can be generated in significant quantities from biomass fermentation and is safer. Consequently, for DAFCs, ethanol is a more pleasant fuel. Nevertheless, the serious problem for commercializing direct ethanol fuel cells (DEFCs) is still the slow reaction kinetics of ethanol electro-oxidation. Many studies of ethanol electro-oxidation have identified the adsorbed intermediates and confirmed the existence of CO intensely adsorbed to the electrocatalyst compared with other species [9,15]. The complete oxidation of ethanol (C_2_H_5_OH) to carbon dioxide consists of 12 electrons released for each C_2_H_5_OH molecule, a C–C connection break, and additional adsorbed middle types that probably poison the platinum (Pt) electrocatalysts, making the reaction more intricate than for CH_3_OH [16]. Thus, some effective anode catalysts should be prepared with adequately high electrochemical activity and CO tolerance for the oxidation of C_2_H_5_OH.

More efforts have been dedicated to preparing electrocatalysts with superior electrochemical activity in ethanol electro-oxidation, revealing the efficiency of adding ceramic oxides to metallic Pt [17]. Based on the findings, some metal oxides, including RuO_2_ [18,19], SnO_2_ [20,21], and WO_3_ [22], can increase the electrocatalytic activity of methanol or ethanol electro-oxidation via synergetic interplay with Pt. Recent approaches using other metal oxides (e.g., CeO_2_ [22], MgO [23], zirconium dioxide (ZrO_2_), and TiO_2_), modified carbon black, and carbon nanotubes have also been developed as the support of Pt electrocatalysts, where the oxide of metals also improves the carbon monoxide (CO) tolerance of the electrocatalysts based on the bifunctional mechanism [23,24,25,26,27,28,29,30,31]. Previous studies found that these fillers increase the electrocatalytic activity of Pt catalysts [20,21]. However, these metal oxides are semiconducting materials with commonly low electric conductivity. Moreover, making extremely dispersed Pt on the surface of oxides is difficult because of the small surface area. For solving this subject and increasing Pt catalyst utilization, other substances with substantial proton and electron conductivity identified as mixed electron-proton conducting articles would be desirable electrocatalyst supports for DAFCs. Furthermore, they provide slight ohmic resistance in both electron and proton conduction, resulting in a highly catalytically active electrocatalyst for ethanol and methanol oxidation. Acidic solids include sulfated metallic oxides, named by several scientists as superacids with excellent proton conductivity. These proton conductor sulfated metallic oxides are relevant in the field of water purifications [32], manufacturing capacitors, batteries, and sensors [33,34,35,36,37,38,39]. Because of their critical role as an electrolyte in fuel cells (FCs) [40,41], they have recently attracted much attention.

One proton conductor with high conductivity is sulfate-treated ZrO_2_, identified as SO_4_-ZrO_2_ solid superacid because the SO_4_ group on the ZrO_2_ surface could increase the hydrophilicity of ZrO_2_ [40,41]. Furthermore, with sulfated ZrO_2_, forming smaller nanoparticles with a larger surface area is more straightforward than with non-sulfated ZrO_2_ [40]. Sulfonating carbon-supported electrocatalysts as mixed protonic and electronic conductors were favorable in increasing the three-phase boundaries [42,43,44,45,46,47].

To enhance the activity of electrocatalyst, one strategy is to explore highly active catalysts with novel carbon material as a support. Graphene nanoplates (GNPs) have gained much attention for their distinctive electrical and mechanical properties in the electrocatalyst support of FCs [48,49]. Graphene-supported Pt or Pt-ruthenium (Ru) nanoparticles exhibited significant catalytic activity for ethanol and methanol electrooxidation [48,49,50,51,52]. These suggest that GNPs could be suitable electrocatalyst supports for FCs.

To the best of our knowledge, no study about Pt/S-ZrO_2_-GNPs electrode in ethanol electro-oxidation reaction in DEFCs has been reported up to now. We propose a hybrid electrocatalyst that integrates the sulfate-treated ZrO_2_ nanocrystals in the vicinity with GNP support and Pt nanoparticles for superior proton and electron conductivity. In this study, the active superacid SO_4_^–2^-ZrO_2_ component has been supported on the GNP surface through the chemical link of proton-conducting sulfonic acid groups onto the GNP surface to form new SO_4_^–2^-ZrO_2_-GNP support for Pt catalysts (Pt/S-ZrO_2_-GNPs) in DEFCs. The resulting Pt/S-ZrO_2_-GNP electrocatalysts were specified by field emission scanning electron microscope (FESEM), X-ray diffraction (XRD), Fourier transform infrared (FTIR), cyclic voltammetry (CV), and impedance curves. The sulfonating effects on the prepared electrocatalysts (Pt/S-ZrO_2_-GNPs) were also explored. The electrodes containing sulfated S-ZrO_2_-GNP powders significantly outperform the unsulfonated ZrO_2_-GNP powder (Pt/ZrO_2_-GNP electrocatalyst). Table 1 shows the comparison between current work and earlier most relevant studies.

Also in the present study, for the synthesis of Pt nanocrystals on S-ZrO_2_-GNP, the polyol process was used in preparing colloidal metal particles [54]. The microwave procedure as a novel technique was adopted in order to avoid the agglomeration of the metal particles at high temperatures. The present microwave-assisted polyol process is simple, practical and effective for rapid synthesis of high dispersed loading Pt-based electrocatalyst. The support graphene is also a microwave-sensitive material, which is believed to play an important role in the acceleration of metal reduction. Especially, the quite short metal reduction process is attractive and interesting from the economic point of view.

## 2. Material and Methods

### 2.1. Materials

Graphite powder (Gr, 99.9999%, 200 meshes) was purchased from Alfa Aesar, Heysham, Lancashire LA3 2XY, UK. Chemical materials, including H_2_PtCl_6_·6H_2_O (40%), ZrOCl_2_.8H_2_O, KMnO_4,_ H_2_SO_4_, 2-propanol, ethanol, (CH_2_OH)_2_, AgNO_3_, and H_2_O_2_ were obtained from Sigma-Aldrich, Chemie GmbH, Eschenstr. 5, Germany. Pt/C (20 wt%) and Nafion^®^ solution (5 wt%, Dupont) were purchased from Fuel Cell Earth Company. CO (99.99%) and N_2_ (99.9995%) gases were provided by Canadian Sigma Inc., 2149 Winston Park Drive, Oakville, ON L6H 6J8, Canada. Milli-Q water was used during the experiment. Polishing kits and glassy carbon (GC) electrodes (d = 5 mm) were provided by Bio-Analytical System (BASi Corporate Headquarters, 2701 Kent Avenue. West Lafayette, In 47906 USA).

### 2.2. Experimental Methods

#### 2.2.1. Preparation of GO and GNPs by Chemical Method

By modifying Hummer’s and Offenman’s method, pristine Gr was converted to graphite oxide under a typical oxidation synthesis process [55]. In ice bath conditions, 1 g of sodium nitrate and 2 g of Gr were poured into 90 mL of concentrated sulfuric acid. Next, 6 g KMnO_4_ was added gradually. That mixture was then stirred at 30 ± 5 °C for approximately 10 h, and 300 mL of deionized water (DI) was added to its contents. Then, it was diluted with 500 mL DI. Next, 5% H_2_O_2_ was dropped into the solution until the color of the slurry transitioned from brown to yellow. The suspension was filtrated through a vacuum Buchner filtration system, and the filtrate was dispersed in DI by an ultrasonic bath. With several centrifugation operations at 11,000 rpm for 25 min, the filtrate was washed out with 1:25 hydrochloric acid dilution and water to a pH value of 7; then, it was dried in an oven at 70 °C for 30 h.

The resulting product was dispersed in DI water and exfoliated to generate graphene oxide (GO) by sonication via a sonotrode (UP400S). After pouring NH_2_NH_2_ into a flask as a reducing agent, H_2_O (hydrazine monohydrate) was dropped into the brown GO nanoplates dispersion. Then, the as-prepared solution was refluxed at 110 °C for 2 h. Consequently, the solution color repeatedly changed to dark black because of the appearance of the GNP dispersion afloat at the solution/air boundary. The dispersion was centrifuged for 10 min at 3500 rpm (SW14, Froilabo, DJB Labcare Ltd, Unit 12, Cromwell Business Centre, Howard Way, Newport Pagnell, Buckinghamshire, MK16 9QS, England) to remove a small amount of precipitate. The supernatants of the GNPs’ dispersal were immediately dried in a freeze dryer to gain the bulk of the graphene nanosheets’ powders.

#### 2.2.2. Preparation of S-ZrO_2_-GNP Support

For preparing S-ZrO_2_-GNPs nanocomposites, GNPs, ZrOCl_2_.8H_2_O, H_2_SO_4_, and NH_3_, were used as starting material, sulfating agent, and precipitating agent. The admixture was brought to a pH of 10 by gradually dropping 28% ammonia aqueous into zirconium oxychloride octahydrate solution (0.20 M) with appropriate quantities of GNPs and stirred for 30 h. The resulting ZrO_2_.nH_2_O sol was washed with purified water using a centrifuge until Cl^-^ were not identified by AgNO_3_, dried at 120 °C for 8 h, and pulverized. The ZrO_2_.8H_2_O and GNP mixture was added to 0.50 M H_2_SO_4_ with vigorous stirring for 20 min, filtered, and dried at 120 °C. Next, the obtained powder was calcinated at 600 °C under airflow for 45 min, and the resulting prepared support was labeled as S-ZrO_2_-GNPs. Then, the S-ZrO_2_-GNP composite and appropriate quantities of chloroplatinic acid hexahydrate were added into 50 mL of Milli-Q water as a suspended solution. For comparison purposes, the ZrO_2_-GNP powder was also prepared with the same procedure without adding H_2_SO_4_.

#### 2.2.3. Polyol Synthesis of Pt/S-ZrO_2_-GNP Nanocomposites by the Aide of Microwave

Pt/S-ZrO_2_-GNP electrocatalysts were constructed by microwave-treating the (CH_2_OH)_2_ solution of H_2_PtCl_6_.6H_2_O precursor salt. As a standard procedure for synthesizing Pt/S-ZrO_2_-GNP with the catalyst loading of 20 wt% Pt, 25 mg $H_{2}{\mathrm{PtCl}}_{6}.6H_{2}O$ was blended with 25 ml ethylene glycol. Then, the pH content of the solution was raised to approximately 12 using NaOH and HCl. Then, 4 mg of Pt/S-ZrO_2_-GNP was homogeneously dispersed in the mixture using an ultrasonic bath. Subsequently, the solution was directly transferred into a microwave oven (in sample cups) for 3 min at 160 °C at 1000 W. The obtained solution was centrifuged at 11,000 rpm at 25 min and the residuals were then washed out with (CH_3_)_2_CO (acetone).

**Figure 1:**
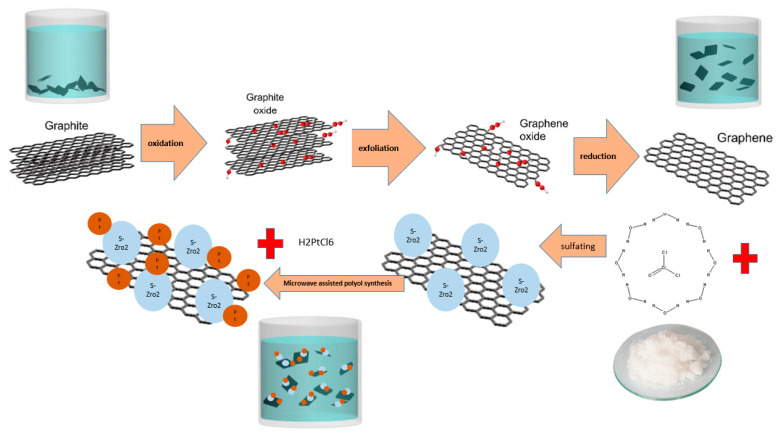
Schematic of experimental methodology for synthesizing Pt/S-ZrO_2_-GNPs.

The resulting precipitate was dried at 100 °C for 24 h under vacuum conditions. For comparison purposes, Pt/ZrO_2_-GNPs nanocomposites with an equal amount of Pt and ZrO_2_ nanoparticles were also prepared by reduction of the $H_{2}{\mathrm{PtCl}}_{6}.6H_{2}O$ precursor salt in ZrO_2_-GNPs mixture (without sulfate) using a microwave for polyol synthesis. A schematic of the experimental methodology used in this study to synthesize Pt/S-ZrO_2_-GNP is illustrated in Figure 1.

#### 2.2.4. Characterization

XRD of Pt/S-ZrO_2_-GNPs was conducted with an Equinox 3000 (IENL France, Head Quarters, INEL, Z.A. C.D. 405) using Cu K$\alpha \;\left(\lambda =0.15406\;\mathrm{nm}\right)$ radiation produced at 40 kV and 30 mA with resolutions of ≤0.1°.

The topography and structure of the graphene nanosheets were studied using an atomic force microscope (AFM, model Nanosurf easy scan2, Nanosurf AG, Graubernstrasse 12, 4410 Liestal, Switzerland) in contact mode. A FESEM description and EDX spectroscopy coupled to an SEM MAG100.00kx with a silicon detector were conducted at 15 kV.

FTIR detections of GO, ZrO_2_/GNPs, and S-ZrO_2_-GNP composites were performed on a WQF-510A/520 FTIR spectrometer No.160 Beiqing Road, Haidian District, Beijing 100095 China.

Electrochemical assessments were performed on a conventional three-electrode cell using Iviumstat potentiostat/galvanostat (vertex, De Zaale 11, 5612 AJ Eindhoven, The Netherlands). Pt wire was used as an auxiliary electrode, Ag/AgCl saturated KCl as a reference electrode, and a GC disk as a working electrode. Before use, the electrode surface was polished via aqueous alumina slurries with sequentially smaller particle sizes between 1 and 0.05 µm on polishing pads. Then, the electrode was sonicated for 15 min in C_2_H_5_OH and DI to eliminate contamination.

The thin film as the working electrode was produced as follows. A catalyst powder with a weight of 10 mg was blended in 1000 µL of isopropanol and Nafion ionomer solution and ultrasonicated for 40 min to produce a fine slurry. Then, the appropriate volume of ink was pipetted onto the surface of the GC disk and dried at 30 °C. A 0.05% Nafion solution was dropped onto the upside of the film to fix the ink on the GC electrode. The electrocatalyst loading on the electrode was $0.4\;{\mathrm{mg}\;\mathrm{cm}}^{-2}$.

## 3. Results and Discussion

### 3.1. Physical Characterization

#### 3.1.1. Topographic Study of GNPs

**Figure 2:**
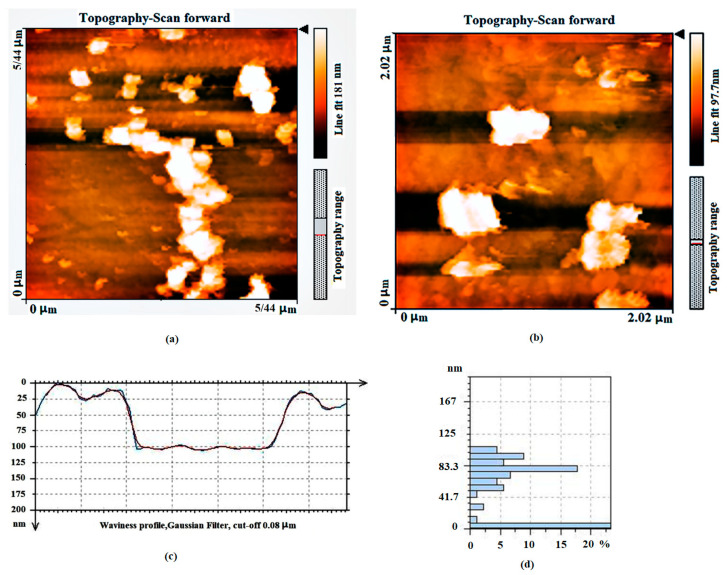
(**a**,**b**) AFM topography images and phase chart of chemical reduced graphene and (**c**) an equivalent height profile, (**d**) pore size distributions.

The resulting GNPs were examined using an AFM to determine the lateral size and thickness. Figure 2 indicates an AFM image and phase chart of GNPs with an equivalent height profile resulting from the chemical reduction of the exfoliated GO along to pore size distribution. The topographic height of the GNPs illustrates few-layered GNPs. Based on the measurements, the thickness of the GNPs was approximately 32–87 nm. From the histogram presented in Figure 2d, it can be seen that 20% of the synthesized graphene nanosheets have a pore size distribution of approximately 1 to 7 nm and the rest from about 8 to 100 nm.

#### 3.1.2. Morphology of ZrO_2_-GNP Support and Pt Nanoparticles

**Figure 3:**
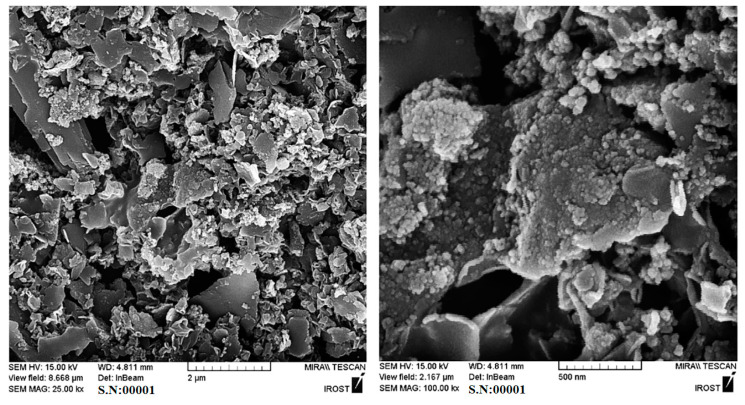
FESEM images of (**a**) GNPs and (**b**) Pt/S-ZrO_2_-GNP electrocatalyst.

Figure 3 depicts the typical FESEM images of the (a) GNPs and (b) Pt/S-ZrO_2_-GNP nanocomposites. Figure 3a also reflects a high mass of chemical-produced GNPs. As illustrated in Figure 3b, the entire surface of GNPs is covered by Pt and S-ZrO_2_ particles. Furthermore, the SEM images indicate that the spherical Pt-S-ZrO_2_ nanoparticles on GNPs are uniform and well-distributed. Since both Pt and S-ZrO_2_ are spherical nanoparticles, it is difficult to exactly identify them in SEM images. However, Pt and S-ZrO_2_ nanocrystals could be identified from the XRD pattern.

#### 3.1.3. Structural Support Specifications

**Figure 4:**
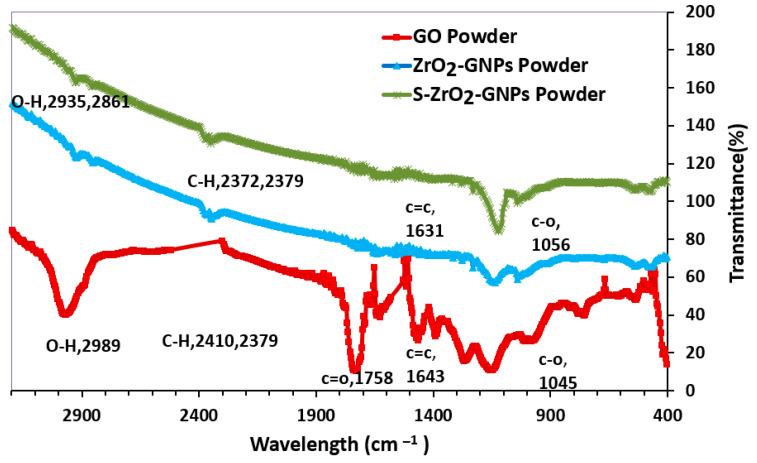
FTIR spectra of GO, ZrO_2_-GNPs, and S-ZrO_2_-GNPs.

Figure 4 illustrates the FT-IR spectra in GO, ZrO_2_-GNPs, and S-ZrO_2_-GNPs. Absorption peaks at 1643 and 2989 ${\mathrm{cm}}^{-1}$ correspond to C = C and C-H modes in GO, while the peaks at 1758 ${\mathrm{cm}}^{-1}$(C = O) are from carbonyl and carboxylic groups and at 1045 ${\mathrm{cm}}^{-1}$ (C–O) are from carbonyl, carboxylic, and epoxy groups, confirming the existence of oxygen-containing functional groups. The peak at 1631 ${\mathrm{cm}}^{-1}$ was reserved in graphene nanosheets, reflecting sp^2^ reconstruction. For the successful removal of C = O, the peak at 1758 ${\mathrm{cm}}^{-1}$ is missing GNPs, revealing the removal of many oxygen groups while transforming from GO to GNPs. The new peaks at 2379 and 2372 ${\mathrm{cm}}^{-1}$ are associated with CH or CH_2_ groups, suggesting that GO was reduced to the GNPs. O-H vibration peaks also occurred at 2861 and 2935 ${\mathrm{cm}}^{-1}$.

Bands at approximately 1130 and 1220–1235 ${\mathrm{cm}}^{-1}$ are associated with the symmetric and asymmetric stretch of the S = O bond. The band at 1040 ${\mathrm{cm}}^{-1}$ is associated with the asymmetric S-O bond. All these bands are relevant to the sulfate bound to the metal oxide in the chelate form [56]. However, for S-ZrO_2_/GNP composite in this study, because S-ZrO_2_ is a nanoparticle, the fine structure splitting of FTIR spectra disappears, and an individual wide band between 1300 and 990 ${\mathrm{cm}}^{-1}$ occurs, caused by the sulfated vibrational modes.

#### 3.1.4. XRD Pattern Characterization

**Figure 5:**
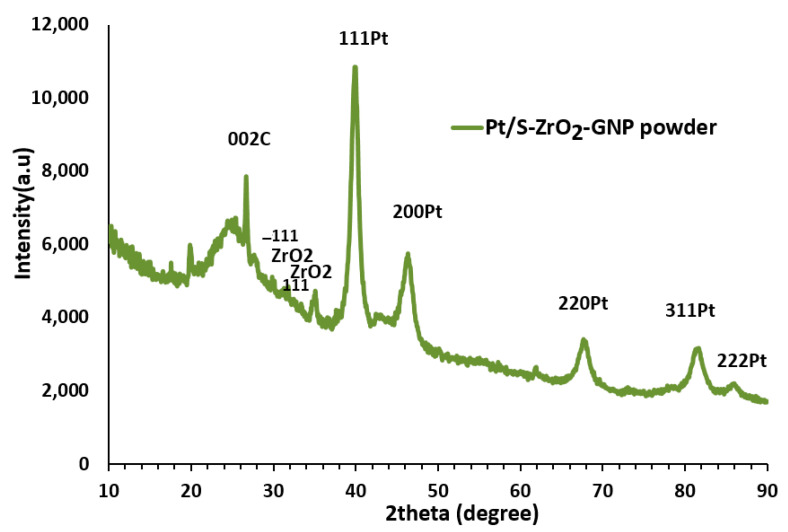
X-ray diffractograms of Pt/S-ZrO_2_-GNP electrocatalyst.

The crystalline lattice patterns of the Pt/S-ZrO_2_-GNP electrocatalyst are depicted in Figure 5, which reveals the existence of both C and Pt. The sharp, thin peak at a 2θ of 26.65° is the feature of the parallel GNP layers in Pt/S-ZrO_2_-GNPs nanocomposite, indicating a highly graphitic and crystalline well-ordered organization of GNPs in the plane of (002). Peaks at the Bragg angles of 85.87°, 81.1°, 67.60°, 46.24°, and 39.855° correspond to the (222), (311), (220), (200), and (111) diffraction peaks of the crystalline plane. All of these peaks suggest the Pt fcc phase. In addition to the specific peaks of the graphite and Pt structure, some other reflectances at a 2θ of 29.95° and 31.69° with planes of −111 and 111 [57,58,59] were established in the electrocatalyst associated with the ZrO_2_ nanoparticles used in the composition of the electrocatalyst.

The mean size of Pt crystals was estimated from the Debby–Scherrer formula using the full width at half maximum (fwhm) of the (111) reflectance. The Pt (111) plane was chosen for Debby’s investigation because it had the maximum intensity. This formula is demonstrated in [60]. XRD results confirmed that treating the nanocrystalline ZrO_2_ with H_2_SO_4_ and the presence of the H_2_SO_4_ did not alter the inherent properties of the electrocatalyst; the mean particle size of the obtained product was approximately 7.66 nm with a d-spacing of 2.26 $\dot{A}$.

#### 3.1.5. Composition of Prepared Electrocatalyst

**Figure 6:**
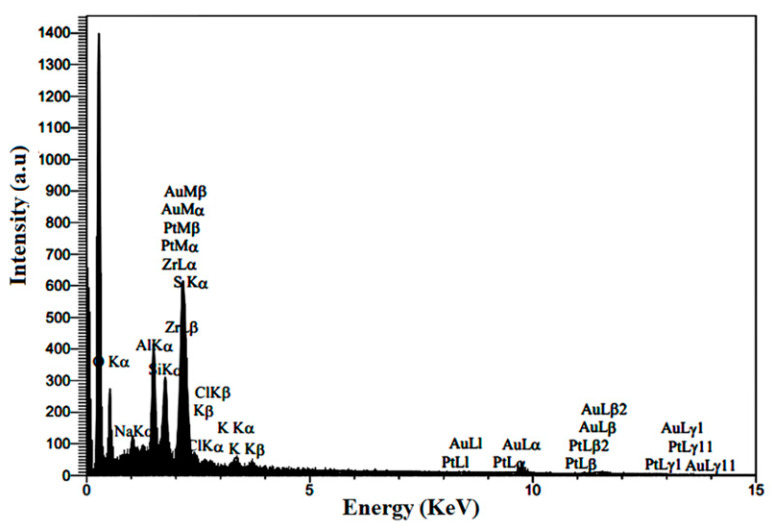
EDX pattern of Pt/S-ZrO_2_-GNP electrocatalyst.

A typical EDS pattern of Pt/ZrO_2_-GNP electrocatalyst is illustrated in Figure 6. This plot indicates that Pt, zirconia, sulfur, and carbon are the primary components of the spectrum—the carbon signal results from the GNPs. In addition to Pt, zirconia, sulfur, and carbon, the components of silicon, Au, and potassium were also observed. The intense peak of silicon could be caused by the Si substrate used in SEM analysis. A small part of Au is from the sputtering unit, and the minimal potassium found in the EDX diagram may be caused by the KMnO_4_ used in GO synthesis. Table 2 presents more information about the chemical composition of the prepared electrocatalyst derived from the EDX pattern. The extent of Pt loading on the composite concerning graphene sheets could be calculated quantitatively as 19.13%, which is near the theoretical magnitude of 20 wt%.

### 3.2. Electrochemical Assessments

#### 3.2.1. Electrochemical Active Surface Area of Electrocatalysts

CV experimentations were conducted in a three-electrode cell using a GC as a working electrode with a specific loading of catalyst enriched with ionomer-blended slurry to estimate the electrochemically active surface area of the products. The working electrode was submerged in a $0.5\;M\;H_{2}SO_{4}$ solution saturated with N_2_, with the voltage sweeping between −0.24 and 1.2 V versus Ag/AgCl at a scan rate of 50$\;{\mathrm{mVs}}^{-1}$. The electrochemical surface area (ECSA) of electrocatalysts was evaluated by computing the hydrogen adsorption/desorption area, using a conversion factor of 210 µC cm^−2^ for polycrystalline Pt [61]. The ECSA was calculated as follows:

(1) $$ECSA=\frac{Q}{\left[Pt\right]\times 0.21}\;$$

where [Pt] is the Pt loading (mg cm^−2^) on the surface of an electrode, Q is the hydrogen adsorption charge (mC cm^−2^), and 0.21 is the required charge for oxidizing a monolayer of H_2_ on brilliant Pt.

**Figure 7:**
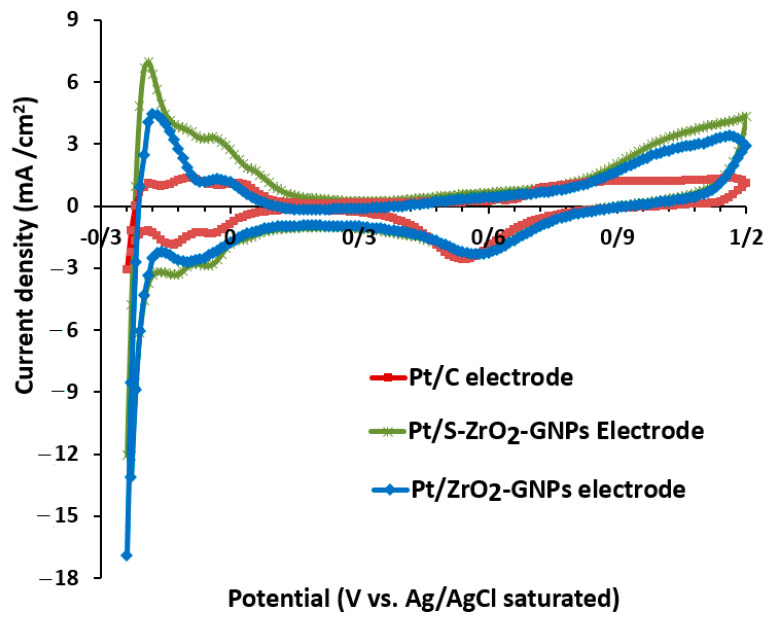
CVs of Pt/S-ZrO_2_-GNPs, Pt/ZrO_2_-GNPs, and Pt/C in $0.5\;M\;H_{2}SO_{4\;}$ solution scan rate: 50${\;\mathrm{mV}\;s}^{-1}$ at 25 °C under N_2_ flux.

The cyclic voltammogram curves of Pt/S-ZrO_2_-GNPs, Pt/ZrO_2_-GNPs, and Pt/C in $0.5\;M\;H_{2}SO_{4}$ are presented in Figure 7. Characteristic hydrogen absorption/desorption peaks between −0.2 and 0.04 V vs. SCE and definite surface oxidation and reduction peaks are evident. The ECSA values of the electrocatalysts computed from the hydrogen desorption charge were 199.1 ${\mathrm{cm}}^{2}.{\mathrm{mg}}^{-1}$ for Pt/S-ZrO2-GNPs, 95 ${\mathrm{cm}}^{2}.{\mathrm{mg}}^{-1}$ for Pt/ZrO_2_-GNPs, and 67.2 ${\mathrm{cm}}^{2}.{\mathrm{mg}}^{-1}$ for Pt/C. Based on this result, the ECSA in the Pt/S-ZrO_2_-GNPs is larger than Pt/ZrO_2_-GNPs; this value for Pt/ZrO_2_-GNPs is greater than that of commercial Pt/C, demonstrating that a superior extent of active sites would be accessible in the hydrogen adsorption and desorption reactions. Consequently, a significant quantity of Pt is employed in the catalyst layer containing sulfated ZrO_2_-GNP support, suggesting that the presence of ZrO_2_ elevates the efficiency of proton exchange membrane fuel cells (PEMFCs).

#### 3.2.2. Performance Estimate of Catalyst through Ethanol Oxidation Reaction (EOR) and Carbon Monoxide (CO) Tolerance

**Figure 8:**
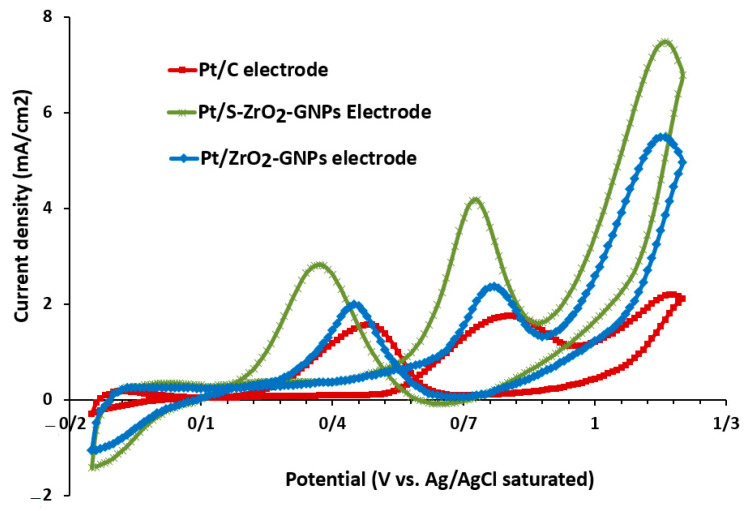
Cyclic voltammograms of ethanol oxidation of Pt/S-ZrO_2_-GNPs, Pt/ZrO_2_-GNP electrodes, and Pt/C electrode in $0.5\;M\;H_{2}SO_{4}+1\;M\;CH_{3}CH_{2}OH$ solution scan rate: 50$\;\mathrm{mV}.s^{-1}$ at 25 °C under N_2_ flux.

Figure 8 illustrates distinctive CV curves of C_2_H_5_OH oxidation on Pt/S-ZrO_2_-GNP electrocatalyst scanned in $0.5\;M\;H_{2}+1\;M\;CH_{3}CH_{2}OH$ at 50 ${\mathrm{mV}\;s}^{-1}$, where CV curves of Pt/ZrO_2_-GNPs and Pt/C were also represented for comparison. The activity of electrocatalysts is examined predominantly using the parameters of peak potential, onset potential, and the magnitude of current density. The anodic forward scan in the voltammogram related to ethanol oxidation and development of Pt-adsorbed carbonaceous intermediates such as COads and CHads, but further electro-oxidation of the adsorbed carbonaceous types to $CO_{2}$ is caused by the backward oxidation peak. C_2_H_5_OH electrooxidation started at roughly 0.53 V for Pt/S-ZrO_2_-GNP, its current peaking at approximately 0.74 V. Reoxidation of ethanol initiated at approximately 0.58 V on the reverse scan and reached a peak current density at approximately 0.37 V, after which severely bonded surface intermediates started blocking the electrocatalyst surface.

The electrochemical manners of Pt/ZrO_2_-GNPs and Pt/C were similar to that of Pt/S-ZrO_2_-GNPs. However, the peak current density of Pt/S-ZrO_2_-GNPs is considerably higher than the two others. The peak current density for Pt/S-ZrO_2_-GNP was approximately 9.45 $\mathrm{mA}.{\mathrm{mg}}^{-1}$ Pt, whereas this parameter for Pt/ZrO_2_-GNPs was approximately 6 $\mathrm{mA}.{\mathrm{mg}}^{-1}$ Pt and Pt/C was only 4.37 $\mathrm{mA}.{\mathrm{mg}}^{-1}$ Pt. The peak potential of the EOR in the forward sweep for all the studied electrocatalysts was virtually the same. However, the onset voltage of EOR on Pt/S-ZrO_2_-GNPs is 0.45 V, Pt/ZrO_2_-GNPs is 0.5 V, and Pt/C reached 0.54 V in the positive-going sweep. This evidence demonstrates that the Pt/S-ZrO_2_-GNP exhibits higher electrocatalytic activity and increased stability for EOR compared with Pt/ZrO_2_-GNPs and Pt/C because of the sulfonating of the ZrO_2_.

Furthermore, the relatively greater IfIb magnitudes on Pt/S-ZrO_2_-GNPs (1.47 vs. 1.20 and 1.11 on Pt/ZrO_2_-GNPs and Pt/C) suggest that ethanol molecules are effectually oxidized on Pt/S-ZrO_2_-GNPs throughout the positive-going voltage scan, producing relatively fewer poisoning types than Pt/ZrO_2_-GNPs and Pt/C. This behavior is likely caused by the synergistic effects of the Pt and ZrO_2_ nanoparticles or the mixed-conducting nature of Pt/S-ZrO_2_-GNP that improves electron and proton transport inside the anode catalyst layer. Consequently, the Pt/S-ZrO_2_-GNP electrode outperformed Pt/ZrO_2_-GNP and Pt/C electrodes in terms of EOR. Pt/S-ZrO_2_-GNP has a much larger ECSA value from its increased hydrophilic properties of the catalyst and increased proton conductivity than Pt/ZrO_2_-GNP and Pt/C, as displayed in Figure 8.

Because CO varieties are the primary poisoning intermediates throughout the EOR, a fine electrocatalyst for EOR would have exceptional CO electrooxidation capability and elevated tolerance, as estimated by the voltammetry of CO stripping. If an electrocatalyst has greater CO electro-oxidation capability and tolerance for CO poisoning, it can oxidize further CO or oxidize CO more rapidly at lowly potentials.

**Figure 9:**
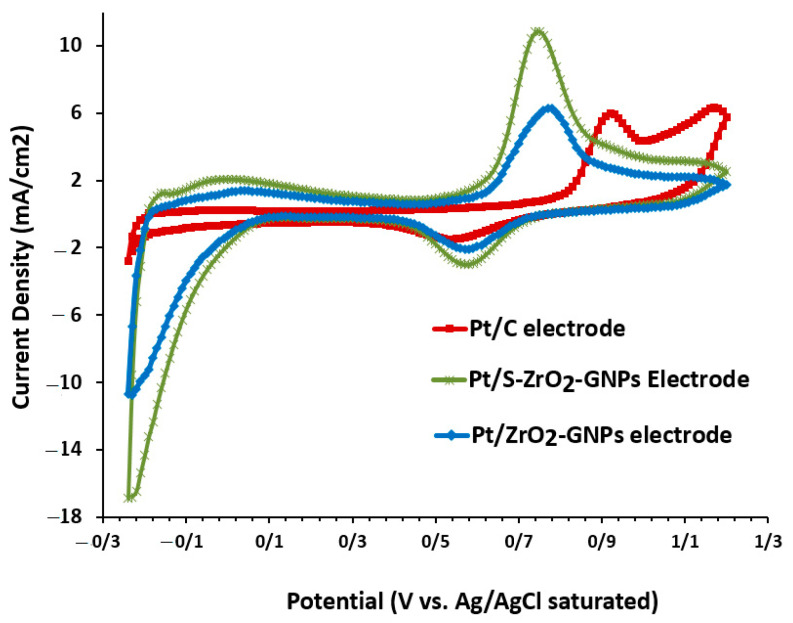
CO stripping results for Pt/S-ZrO_2_-GNPs, Pt/ZrO_2_-GNPs, and Pt/C electrodes in $0.5\;M\;H_{2}SO_{4\;}+1\;M\;CH_{3}CH_{2}OH$ solution, scan rate: 50${\;\mathrm{mV}\;s}^{-1}$ at 25 °C.

The voltammogram curves of CO stripping on Pt/S-ZrO_2_-GNPs, Pt/ZrO_2_-GNPs, and Pt/C are depicted in Figure 9, accomplished by electrooxidation of pre-adsorbed CO. Considerable differences in the peak potential and onset voltage for CO electrooxidation between the electrocatalysts comprising ZrO_2_ and original Pt were observed. Another observation from the CO stripping revision correlated with the CO electrooxidation onset potential, which is applied to specify the easiness of CO electrooxidation. The onset voltage of CO electrooxidation on Pt/S-ZrO_2_-GNPs was 0.52 V, which is approximately 0.07 V lower than that recorded for the Pt/ZrO_2_-GNP electrode and 0.21 V lower for Pt/C, demonstrating the practical function of ZrO_2_-GNPs and sulfonation of ZrO_2_-GNPs for CO electrooxidation. The peak voltage of CO electrooxidation on Pt/S-ZrO_2_-GNP was 0.76 V, which was decreased by 0.03 V and 0.17 V in comparison with 0.79 V for Pt/ZrO_2_-GNP and 0.93 V for Pt/C. Moreover, the electrooxidation current density of the COads varieties on the Pt/S-ZrO_2_-GNPs is approximately 10.86 $\mathrm{mA}.{\mathrm{cm}}^{-2}$, which is considerably greater than that of Pt/ZrO_2_-GNP and Pt/C (6.25 and 5.91 $\mathrm{mA}.{\mathrm{cm}}^{-2}$), indicating that the Pt/S-ZrO_2_-GNP electrode is relatively tolerant to CO poising compared to the Pt/ZrO_2_-GNPs and Pt/C electrode.

$Q_{CO}$, the peak charge, is relevant to the reaction

(2) $$Pt-CO+H_{2}O\to Pt+CO_{2}+2H^{+}+2e^{-}$$

The charge $Q_{CO}$ (mC cm^−2^) is used to compare the active surface area (ESA) of the electrocatalyst, computed as follows:

(3) $$ESA_{CO}=\frac{Q_{CO}}{0.484\times \left[Pt\right]}\;$$

where the value 0.484 denotes the charge density requisite to oxidize a monolayer of CO on bright Pt [62,63].

The active surface area magnitudes estimated from the CO electrooxidation surface area (ESA_CO_) were 99.17, 78.18, and 13.55 ${\mathrm{cm}}^{2}.{\mathrm{mg}}^{-1}$ for Pt/S-ZrO_2_-GNP electrode, Pt/ZrO_2_-GNPs, and Pt/C. The greater ESA_CO_ of Pt/S-ZrO_2_-GNP electrocatalyst directs greater Pt utilization in the related electrocatalyst. Thus, the Pt/S-ZrO_2_-GNP catalyst could provide improved activity over the CO oxidation. This can be explained by the electrooxidation of CO comprising adsorbed OH types with the adsorbed CO. The SO_4_^–2^ group on the ZrO_2_ surface improved the hydrophilicity of ZrO_2_ and the additional dissociative hydroxyl of H_2_O absorbed on the solid superacid than that of the non-sulfated ZrO_2_. Consequently, sulfate-treated ZrO_2_ could much more simply transform COads to carbon dioxide by the hydroxyl on its surface based on the bifunctional mechanism, leaving the active sites of Pt for the additional electrochemical reactions [28,31].

#### 3.2.3. Electrochemical Impedance Spectroscopy (EIS) Investigations of the Electrodes

The electrochemical impedance spectroscopy (EIS) studies of the electrodes were performed at 0.05 V versus SCE by scanning frequencies in the range of 0.1 to 100E+3 Hz with 67 decades and an alternating sinusoidal signal of 0.05 V peak to peak. In those studies, the prepared electrodes were subjected to a nitrogen-saturated solution of 0.5 M H_2_SO_4_ and 1.0 M $CH_{3}CH_{2}OH$. All electrochemical assessments were accomplished below 25 °C.

**Figure 10:**
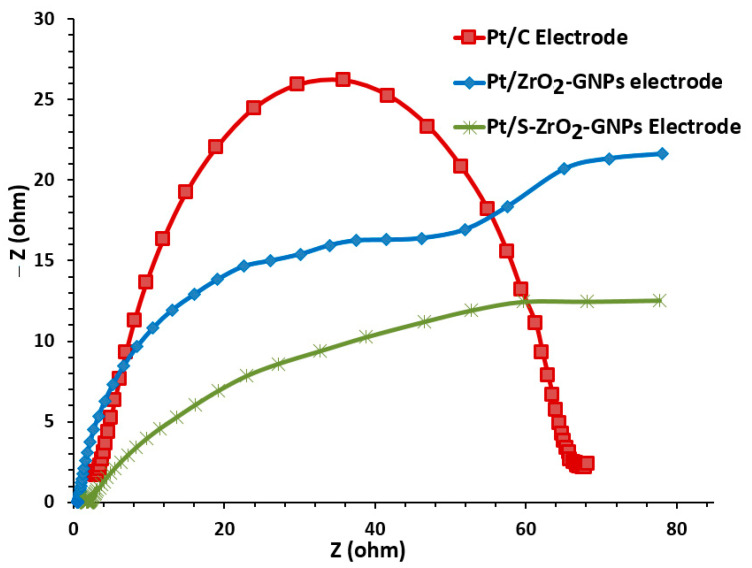
EIS diagram in Nyquist form of Pt/S-ZrO_2_-GNPs, Pt/ZrO_2_-GNPs, and Pt /C electrodes in 0.5 M H_2_SO_4_ + $1M\;CH_{3}CH_{2}OH$ at 25 °C under N_2_ flux.

The related Nyquist plots are presented in Figure 10. Both the Nyquist plots of Pt/S-ZrO_2_-GNPs and Pt/ZrO_2_-GNPs display similar features, including a depressing arc in the high-frequency zone, whose diameters linked with the charge transfer resistance representing the electrocatalytic activity for alcohol oxidation reaction and a relatively straight lines in the low-frequency zone that relates to diffusion-limiting of the electrocatalyst. The diameter of the arc for Pt/S-ZrO_2_-GNP electrocatalyst compared with Pt/ZrO_2_-GNP electrode is significantly depressed, suggesting simple ethanol electrooxidation. The addition of sulfate-treated ZrO_2_-GNPs to Pt seems to diminish the charge transfer resistance more than that of non-sulfated ZrO_2_-GNPs, demonstrating an increased ethanol oxidation activity by Pt/S-ZrO_2_-GNPs. These results are caused by the improved electron and proton conductivity of the Pt/S-ZrO_2_-GNP electrode. The contraction in arc diameter in Nyquist charts designates the developed catalytic activities of Pt nanoparticles supported with S-ZrO_2_/GNP composite, consistent with the electrocatalytic activities deduced from CV measurements.

**Figure 11:**
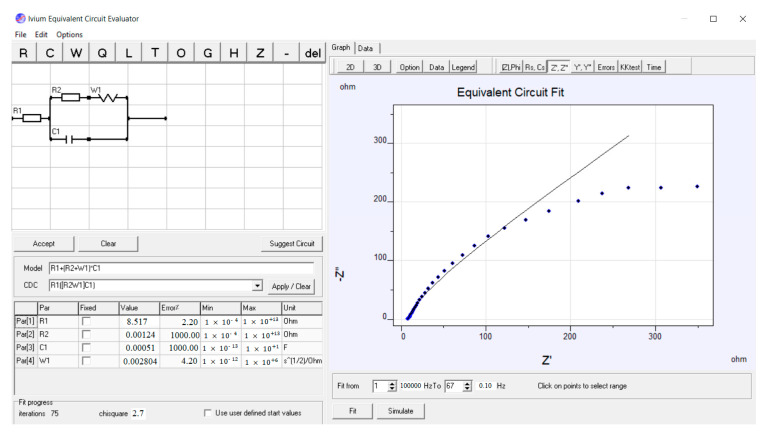
EC of fitted EIS data of Pt/S-ZrO_2_-GNPs in $0.5\;M\;H_{2}SO_{4}\;$+$\;1\;M\;CH_{3}CH_{2}OH$ solution.

The fundamental aspects of the impedance assessments can be described using the equivalent circuit (EC) in Figure 11. This circuit includes the sum of electrode and electrolyte ohmic resistance (R1) and charge transfer resistance (R2), which controls the electron transfer kinetics of electroactive types at the electrode interface, diffusion-limiting Warburge (W1) of the electrocatalyst parallel to the constant phase element related to double layer capacity (C1). The experimental impedance data are represented in the diamond, while the solid line signifies a theoretical fit obtained using the EC model displayed in Figure 11.

## 4. Conclusions

In this study, high-density GNPs were prepared by modifying Hummer’s and Offeman’s method. A S-ZrO_2_-GNP was prepared successfully by treating sulfonic acid groups onto the surface of ZrO_2_-GNPs via chemical methods. A Pt/S-ZrO_2_-GNP-modified GC electrode was constructed successfully by novel microwave-assisted polyol synthesis method and its physical and electrochemical properties with special characteristics were observed. These properties include a successful dispersion of catalyst nanoparticles on support, lower CO electro-oxidation peak potential (0.76 vs. 0.79 and 0.93 V), much higher ethanol electro-oxidation current density (9.45 vs. 6 and 4.37 $\mathrm{mA}.{\mathrm{mg}}^{-1}$ Pt) and low charge transfer resistance in the presence of ethanol in compression to Pt/ZrO_2_-GNPs and commercial Pt/C. The higher electrochemical activity of Pt/S-ZrO_2_-GNPs can be attributed to the CO poising of the platinum being lowered by ZrO_2_-GNP sulfonation and increased reaction kinetics as observed from EIS measurements. These findings imply that Pt/S-ZrO_2_-GNPs is a promising electrocatalyst for applications of the anode with greater EOR activity and elevated CO tolerance in higher-performance DEFCs.

## Figures and Tables

**Table 1:** Comparison between current work and earlier most relevant studies.

Electrocatalyst and Support Material	Synthesis Technique	Fuel Type	Electrochemical Assessment Methods	Results	Reference
Pt/S-ZrO_2_/MWCNT	NaBH4 reduction procedure	Methanol	(1) Cyclic voltammetry (2) Impedance spectroscopy (3) Polarization test	(1) The Pt–S-ZrO_2_ MWCNT exhibited higher MOR activity than Pt-ZrO_2_/MWCNT and Pt/C. (2) The charge transfer resistance of Pt–S-ZrO_2_/MWCNT was smaller than Pt-ZrO_2_/MWCNT. (3) Pt–S-ZrO_2_/MWCNT maintained the highest current density among all the catalysts.	[31]
Pt/S-TiO_2_/MWCNT	NaBH4 reduction procedure	Ethanol	(1) Cyclic voltammetry (2) Electrochemical impedance spectroscopy (3) Chronoamperometry	(1) Pt–S-TiO_2_/MWCNT had shown higher catalytic activity for EOR compared with Pt/TiO_2_/MWCNT and Pt/C. (2) Pt–S–TiO_2_/MWCNT decrease the charge transfer resistance than Pt/TiO_2_/MWCNT and Pt/C. (3) the current density of EOR on the Pt–S–TiO_2_/ MWCNT and Pt–TiO2/MWCNT electrode decreases slowly than Pt/C	[28]
Pt/ZrO_2_/C	NaBH4 reduction procedure	Ethanol	(1) Cyclic voltammetry (2) Electrochemical impedance spectroscopy (3) Tafel plot	(1) Pt–ZrO_2_/C catalyst had higher peak current density and lower peak potential than Pt/C. (2) The charge transfer resistance of Pt–ZrO_2_/C was smaller than Pt/C (3) The similar Tafel slopes obtained at the two catalysts of Pt–ZrO_2_/C and Pt/C	[23]
Pt/TiO_2_/CNT	Sol–gel and ethylene glycol reduction method	Ethanol	(1) Cyclic voltammetry (2) CO stripping voltammetry (3) Chronoamperometry	(1) Pt–TiO_2_/CNTs catalysts have higher electrocatalytic activity and CO-tolerance for EOR than Pt/C and Pt/CNTs catalyst in acid. (2) Lowest peak potential for CO electro-oxidation on Pt–TiO_2_/C catalysts was obtained. (3) The ethanol oxidation current density at Pt–TiO_2_/CNTs electrode was higher than that at Pt/CNTs and Pt/C electrode though the current decay with time	[29]
Pt/S-CNT	Ethylene glycol reduction method by refluxing	Methanol	(1) Cyclic voltammetry (2) Electrochemical impedance spectroscopy (3) Long-term stability test	(1) Pt/sulfonated-CNTs catalysts had high electrocatalytic activity and excellent electrocatalytic performance for the DMFC. (2) The electrocatalytic oxidation of methanol on Pt/sulfonated-CNTs/GC electrode is a diffusion-controlled process. (3) The Pt/sulfonated-CNTs/GC electrode had extraordinarily high electrocatalytic stability and storage properties.	[53]
Pt/S-ZrO_2_/GNPS	Microwave-assisted polyol synthesis method	Ethanol	(1) Cyclic voltammetry (CV) in terms of electrochemical surface area and ethanol electro-oxidation (2) CO stripping voltammetry (3) Electrochemical impedance spectroscopy	(1) Pt/S-ZrO_2_-GNPs electrocatalyst exhibited high catalytic activity for the EOR in terms of electrochemical surface area with respect to Pt/ZrO_2_-GNPs and Pt/C. (2) Pt/S-ZrO_2_-GNP-modified GC electrode exhibited lower CO electro-oxidation peak potential, much higher EOR current density in compression to Pt/ZrO_2_-GNPs and Pt/C. (3) EIS results showed, the Pt/S-ZrO_2_-GNPs electrocatalyst has a low charge transfer resistance than Pt/ ZrO_2_-GNPs and Pt/C in the presence of ethanol.	Current work

**Table 2:** Composition of Pt/S-ZrO_2_-GNP composite powder (quantitative results).

Element	Line	Int	W%	A%
C	Ka	452.7	60.14	83.42
O	Ka	73.2	10.29	10.71
Na	Ka	47.3	0.95	0.69
Al	Ka	183.9	2.43	1.50
Si	Ka	140.5	1.77	1.05
S	Ka	17.8	0.3	0.15
Cl	Ka	14.7	0.28	0.13
K	Ka	25.5	0.57	0.24
Zr	Ka	52.8	1.35	0.25
Pt	Ka	9.5	19.13	1.62
Au	Ka	1.8	2.8	0.24
			100	100

## Data Availability

Not applicable.
